# Caught off guard and beaten: The Ukraine war and food security in the Middle East

**DOI:** 10.3389/fnut.2023.983346

**Published:** 2023-02-21

**Authors:** Mohammad Al-Saidi

**Affiliations:** Department of International Affairs and Center for Sustainable Development, College of Arts and Sciences, Qatar University, Doha, Qatar

**Keywords:** food security, Middle East, Ukraine war, global food crisis, Russia, grain

## Abstract

The Ukraine war has led to a severe global food crisis due to complex supply disruptions and price increases of agricultural inputs. Countries of the Middle East have been directly affected because of their high dependence on food imports from Russia and Ukraine. Furthermore, this food crisis comes at times of high baseline vulnerability due to the compound impacts of COVID-19, repeated food shocks, and weakened states due to political-economic difficulties. This paper provides a detailed analysis of the food-related vulnerability of Middle Eastern countries in the wake of the Ukraine war. It contextualizes the varying impacts of this crisis in the region, and highlights country-level response strategies. The analysis shows a concerning and deepened crisis in the case of highly exposed and politically fragile countries with weakened food sectors; e.g., Lebanon, Sudan, and Yemen. Political-economic instabilities, limited domestic agriculture, and the lack of reliable grain reserves have aggravated the current food crisis in some countries. At the same time, indigenous short-term responses related to regional aid and cooperation have emerged, particularly in the Gulf countries, which have witnessed soaring revenues from higher energy prices. Alongside more regional frameworks for collaboration on food security, future action to mitigate such food crises should include the strengthening of local sustainable agriculture, storage capacities, and grain procurement strategies from international suppliers.

## 1. Introduction

The Ukraine war marks a new era in international diplomatic and economic relations, with major anticipated reconfigurations of trade flows. Not only are the direct disruptions to production in violence-ridden areas a matter of concern, but the war has also been accompanied by sanctions and boycotts causing major value-chain disruptions. In fact, although the effects take time, trade has always been part of the collateral damage of wars, with significant associated costs (1, 2). Furthermore, war-related impacts such as decoupling from the global economy, imposed sanctions, and (to some degree) consumer-led boycotts can cause further trade damage (3, 4). In the case of the Ukraine war, the impacts on global trade and economic relations have been immediate in terms of downgraded global growth (estimated at 3.5% instead of the usual level of above 4% for 2022) (5). The consequences go far beyond the European continent as regions such as Latin America and Africa have felt the economic impact through inflated commodity prices and financial volatility (6, 7).

The long-term consequences for energy trade with Europe after the Ukraine war has been a key subject of debate. However, in the short term, the war has already resulted in serious food security concerns for highly vulnerable regions (8). It has immediately caused major risks and shortfalls; e.g., related to production, trade flows, and prices of food commodities, since Ukraine and/or Russia have been among the top three global producers of wheat, maize, rapeseed, sunflower seeds, and sunflower oil (9). Furthermore, Russia ranks very high globally in the production of key fertilizers (ibid.). Therefore, there have been dire warnings of food insecurities as a result of the Ukraine war. With less grain and fertilizer available due to the war, the global food supply is threatened. Between 2016 and 2021, Ukraine and Russia produced more than 50% of the world’s supply of sunflower seed, 19% of the world’s barley, and 14% of its wheat (10), while they accounted for ca. 30% of global wheat exports (11, 12). With at least 50 countries depending on Russia and Ukraine for 30% or more of their wheat supply, a global food crisis has been triggered, exacerbated by higher energy prices, as was also the case in the recent crises of 2007–2008 and 2010–2012 (13). The soaring energy prices affected fertilizer costs (natural gas is used in fertilizer production) and thus restrained local production worldwide, including in Europe (14–16). The Black Sea Grain Initiative (BSGI) signed in July 2022 to allow some grain exports from Ukraine has alleviated some impacts of the Ukraine war on food security, particularly easing pressures on markets for grains (17). However, as of January 2023, the BSGI is still fragile due to restrictions of shipments while an enduring global food crisis is still persistent (18).

The complexity of the food crisis caused by the Ukraine war necessitates detailed assessments of regional vulnerabilities. The Middle East is considered to be one of the worst-hit regions since several countries in the region are listed among the countries most dependent on agri-food commodities from Ukraine and Russia; e.g., in order of dependence, Turkey, Egypt, Sudan, Tunisia, Morocco, and Saudi Arabia (19). Other reports indicate that other countries such as Lebanon, Yemen, and Jordan are particularly vulnerable, and highlight special cases of existential threats; e.g., for Egypt (20). Within this focus on the repercussions of the Ukraine war on food security in Middle Eastern countries, there is a need to go beyond rapid assessments of past dependence to analyze the vulnerability contexts of these countries. This paper presents such a contextualization of the food security impacts of the Ukraine war. Here, food security is defined in accordance to the United Nations (UN) as all people, at all times having “physical and economic access to sufficient safe and nutritious food that meets their dietary needs and food preferences for an active and healthy life” (21).

There is so far no knowledge regarding the impacts of the Ukraine war within the context of repeated shocks from COVID-19 and economic or political crises in the Middle East. To address this, this paper aims to provide a more detailed analysis of the vulnerability of the Middle East to food insecurity in the aftermath of the Ukraine war. It contextualizes this food crisis within country-level vulnerabilities and recent political-economic shocks. This paper’s analysis focuses on access aspects to food in the wake of the Ukraine crisis, with an emphasis on stable crops. Through identifying high-risk countries and possible mitigation strategies, this paper shows the impacts of the food security crisis on Middle Eastern countries, and how these impacts happen at different speeds. It also illustrates indigenous adaptation pathways using intraregional cooperation mechanisms. While this paper focuses on access to stable crops, it does not tackle nutrition and dietary aspects; for example, it does not look into horticultural produce and animal-source food which are also imported into the Middle East.

## 2. Recurrent food supply shocks: from COVID-19 to the Ukraine war

In 2022, many developing countries, particularly those in Africa and the Middle East, have been shaken by the impacts of the COVID-19 pandemic. In this year, it is estimated – before the Ukraine war – that around 44 million people in 38 countries are threatened by hunger (22). In the Middle East and North Africa, the number of food-insecure people has increased dramatically, with one in three people in 2020 with no access to adequate food, an increase of 10 million people from 2019 (23). Besides COVID-19, several countries in the Middle East (defined broadly in this paper to include countries of the Arab League as well as Turkey and Iran) are suffering from protracted conflicts (e.g., Yemen, Syria, Libya), increased political instability (e.g., Lebanon, Sudan, and Tunisia), or the aftermath of hard economic reforms (e.g., the impacts of structural reforms in Egypt). The Middle East has been one of the world’s major cereal-importing regions, particularly of wheat, while food supply problems – limited yields or increased prices–of grain such as wheat from Ukraine and Russia have historically affected food security in this region (24). For example, interruptions of grain exports from Russia, Ukraine or Kazakhstan due to harvest failure or export restrictions immediately resulted in soaring costs for the food subsidy systems in major dependent countries (25).

Meanwhile, the negative impacts of the COVID-19 crisis on food security in Middle Eastern countries have been well-documented in the academic literature. COVID-19 has resulted in serious disruptions to the food value chain, with grain export restrictions during the pandemic, together with locusts destroying crops and causing price hikes, and food insecurity across many regions including parts of the Middle East (26). Countries located in the Sahel region have been particularly vulnerable during the pandemic, with COVID-19 resulting in weakened food sectors (27). COVID-19 has forced a re-evaluation of the water–food–trade link within the water–energy–food nexus, and reignited debates regarding self-sufficiency and the expansion of the local food production, even in arid regions (28). COVID-19 might have increased isolationist voices in many of the world’s regions, in contrast to voices advocating for the strengthening of the resilience of international trade. However, self-sufficiency in food products is an illusionary strategy in the arid region of the Middle East, and past strategies in this regard–e.g., in countries of the Gulf Cooperation Council (GCC) – have failed (29).

Prior to COVID-19 and the current Ukraine-related crisis, the Middle East had only recently emerged from years of recurrent food shocks. Early this century, Middle Eastern countries suffered from unreliable imports and began investing in reserves and the acquisition of foreign agricultural land (30, 31). Moreover, many countries of the region (e.g., Syria, Yemen, South Sudan, and Somalia) have suffered from serious conflict-related economic shocks, thus jeopardizing food and nutrition security (32). As an additional aggravator of baseline food supply vulnerability, climate-related shocks are causing food insecurity. In particular, several types of droughts (hydrological, meteorological, and agricultural) have affected the performance of the food sectors in the Middle East in recent decades (33). With the earlier-mentioned impacts of COVID-19, in the year 2021, serious supply bottlenecks existed, with the price of wheat and barley increasing by 31%, and rapeseed oil and sunflower oil by more than 60% (12). These factors combined indicate an alarming trend of increased vulnerability of food supply systems in the Middle East, which has been aggravated by the Ukraine crisis, thus bringing the region close to a worst-case scenario of food insecurity. The BSGI seems to have avoided this worst-case scenario for the Middle East and North African (MENA) region since the region has received a significant amount of the grain exports under this deal, e.g., 42% of Ukrainian grain exports between August and October 2022, and 28% of its corn exports for the same period (34). However, the faith of the BSGI was uncertain in late 2022 as Russia threatened to leave the agreement and still restricted some shipments in early 2023 (18). Any halt of the BSGI would severely set back progress toward mitigating the food crisis in the Middle East (35). Besides, the level of imports from Ukraine in 2022 was far below the 2021 level for the important grain of wheat (34). In fact, as of January 2023, 17.8 million tons of grains were shipped from Ukraine, of which 46% were corn and 28% wheat, with China, Spain, Turkey, Italy, and the Netherlands as the main destinations (36). Therefore, the BSGI is geared toward containing prices and stabilizing markets rather than averting famine (17). As this paper will explain in the next sections, the repercussions of the Ukraine crisis on the Middle East’s food security have been profound in 2022 and with far-reaching impacts beyond.

## 3. Methodology and data

In order to assess and contextualize the food-related vulnerabilities of Middle Eastern countries in the wake of the Ukraine water, the analysis in this paper is carried out in two steps. Firstly, the relative level of vulnerability to supply risks from Ukraine and Russia is determined. For this, trade data obtained from UN Comtrade are initially used (aggregated for the last five years, 2016–2020) to determine the biggest importers from Russia and Ukraine in key food commodity categories, and also to determine dependence levels of Middle Eastern countries on imports from Russia and Ukraine in some important food commodity categories. Later, in order to categorize the vulnerability of Middle Eastern countries, the dependence indicators are combined with data from the Global Food Security Index (GFSI) published by the Economist. The GFSI is a composite index using indicators in four categories: affordability, availability, quality and safety, and natural resources and resilience. In our analysis, the data provided by the GFSI on the baseline vulnerability of Middle Eastern countries to increased risks due to the Ukraine war, together with the dependence indicators, allow information to be extracted on which Middle Eastern countries are particularly vulnerable during this crisis.

Secondly, using the categorization of Middle Eastern countries in terms of relative vulnerability related to the food insecurity crisis caused by the Ukraine war, a country-level case analysis is carried out. For this, the paper uses the recent academic literature, announcements from international organizations, and media reports on country-level adaptation measures in order to present the vulnerability contexts at the country level. As for academic literature on the impacts of the Ukraine crisis on food security in the Middle East, this study does not present a full literature review due to the lack of studies on this recent topic. For example, a scopus-based search was conducted in January 2023 using the keywords “food security” and “Ukraine” in either in the title, abstract or keywords of the publications, together with any of the following keywords: Middle East, MENA, North Africa or the country name (see Table 2). The resulting dataset was only 11 documents, 7 of which from 2022 or later, and only 3 on a Middle Eastern country (2 on Lebanon and 1 on the UAE). This small number of papers was used in the analysis, but it largely relied on earlier studies known to the author on the vulnerability context, and the earlier-described types of documents available through online search related the current conflict. Together, these studies and documents elaborate the contexts that are related to the performance of the local food sector in response to the recent food-related shocks. In addition, through analyzing the cases, a particular emphasis is placed on required actions and short- to medium-term adaptation strategies to be carried out by the Middle Eastern countries and through the mechanisms of regional or international cooperation. Such an analysis is later synthesized into larger regional lessons learnt from contextualizing the current crisis within recent developments affecting vulnerability in the Middle East due to compounded health and political-economic shocks.

**TABLE 1:** The biggest importers of key food commodity categories from Ukraine and Russia (2016–2020).

Rank^a,^ ^b^	Cereals^1^		Milling industry^2^			Oil seeds^3^			Fats and oil^4^		
**Biggest importers from UKRAINE 2016–2020 (amount in billion USD included after country name)**											
**Total**	**World**	**38.87**	**Tot**	**World**	**0.86**	**Tot**	**World**	**9.96**	**Tot**	**World**	**23.56**
**1**	Egypt	4.69	**1**	China	0.08	**1**	Turkey	1.65	**1**	India	7.43
**2**	China	4.18	**2**	Rep. of Moldova	0.07	**2**	Germany	1.48	**2**	China	3.32
**3**	Spain	2.86	**3**	United Arab Emirates	0.07	**3**	Belgium	1.14	**3**	Netherlands	1.91
**4**	Netherlands	2.35	**4**	Angola	0.06	**4**	Netherlands	0.85	**4**	Spain	1.61
**5**	Indonesia	2.23	**5**	Israel	0.04	**5**	Egypt	0.78	**5**	Italy	1.29
**6**	Turkey	1.63	**6**	Indonesia	0.04	**6**	France	0.55	**6**	Iraq	0.95
**7**	Bangladesh	1.52	**7**	State of Palestine	0.04	**7**	Belarus	0.51	**7**	Poland	0.78
**8**	Italy	1.38	**8**	Poland	0.03	**8**	Iran	0.44	**8**	France	0.52
**9**	Saudi Arabia	1.38	**9**	Singapore	0.03	**9**	Poland	0.41	**9**	Iran	0.51
**10**	Tunisia	1.38	**10**	Brazil	0.02	**10**	Italy	0.26	**10**	Egypt	0.41
**11**	Israel	1.24	**11**	Somalia	0.02	**12**	Lebanon	0.22	**13**	Turkey	0.37
**12**	Libya	1.14	**14**	Turkey	0.01	**14**	Israel	0.14	**14**	United Arab Emirates	0.34
**14**	Iran	1.1	**15**	Egypt	0.01	**18**	United Arab Emirates	0.09	**15**	Saudi Arabia	0.22
**15**	Morocco	0.97	**18**	Saudi Arabia	0.01	**27**	Algeria	0.02	**16**	Lebanon	0.22
**20**	Lebanon	0.6				**29**	Tunisia	0.02	**19**	Jordan	0.14
**22**	Algeria	0.49							**20**	Sudan	0.12
**25**	Yemen	0.39							**23**	Oman	0.12
**28**	Jordan	0.23							**25**	Israel	0.08
**31**	Mauritania	0.17							**33**	State of Palestine	0.05
**38**	United Arab Emirates	0.12							**37**	Qatar	0.04
									**57**	Djibouti	0.02
**43**	Sudan	0.01							**65**	Kuwait	0.02
**46**	Djibouti	0.01							**69**	Yemen	0.02
**47**	Qatar	0.01							**79**	Syria	0.01
**50**	Oman	0.01							**92**	Bahrain	0.01
**53**	Kuwait	0.01									
**Biggest importers from RUSSIA 2016–2020 (amount in billion USD included after country name)**											
**Total**	**World**	**40.82**	**Tot**	**World**	**1.42**	**Tot**	**World**	**4.58**	**Tot**	**World**	**14.92**
**1**	Egypt	7.4	**1**	Norway	0.17	**1**	China	1.61	**1**	China	2.47
**2**	Turkey	6.09	**2**	China	0.16	**2**	Turkey	0.74	**2**	Turkey	1.79
**3**	Bangladesh	1.91	**3**	Belarus	0.13	**3**	Belarus	0.55	**3**	Egypt	1.03
**4**	Iran	1.79	**4**	Kazakhstan	0.1	**4**	Belgium	0.36	**4**	Iran	0.96
**5**	Saudi Arabia	1.68	**5**	USA	0.09	**5**	Bulgaria	0.17	**5**	Uzbekistan	0.84
**6**	Sudan	1.32	**6**	Turkey	0.07	**6**	Kazakhstan	0.14	**6**	Kazakhstan	0.8
**7**	Nigeria	1.29	**7**	Uzbekistan	0.06	**7**	Latvia	0.13	**7**	Norway	0.79
**8**	Azerbaijan	1.16	**8**	Ukraine	0.06	**8**	Poland	0.11	**8**	Algeria	0.75
**9**	Viet Nam	1.07	**9**	Azerbaijan	0.06	**9**	Mongolia	0.11	**9**	India	0.58
**10**	Yemen	1.05	**10**	Georgia	0.06	**10**	Germany	0.09	**10**	Belarus	0.53
**11**	Lebanon	0.84	**24**	Israel	0.01	**12**	Iran	0.06	**13**	Sudan	0.25
**12**	United Arab Emirates	0.71	**25**	United Arab Emirates	0.01	**21**	Egypt	0.02	**15**	Saudi Arabia	0.23
**15**	Israel	0.61	**29**	Syria	0.01				**18**	Lebanon	0.21
**20**	Jordan	0.52							**26**	Tunisia	0.13
**22**	Morocco	0.47							**30**	Israel	0.07
**23**	Libya	0.45							**32**	Syria	0.06
**26**	Oman	0.36							**37**	Morocco	0.03
**42**	Syria	0.15							**44**	Jordan	0.02
**51**	Qatar	0.12							**46**	United Arab Emirates	0.01
**61**	Algeria	0.01							**49**	Oman	0.01
**62**	Mauritania	0.01									
**69**	Kuwait	0.01									
**73**	Djibouti	0.01									

(a) All data represent values of Russian exports to indicated countries in billion USD. The food commodity category are as follows: (1) Cereals = Commodity Code 10: Cereals; (2) Milling industry = Commodity Code 11: Products of the milling industry; malt, starches, inulin, wheat gluten; (3) Oil seeds = Commodity Code 12: Oil seeds and oleaginous fruits; miscellaneous grains, seeds and fruit, industrial or medicinal plants; straw and fodder; (4) Commodity Code 14 = Animal or vegetable fats and oils and their cleavage products; prepared animal fats; animal or vegetable waxes.

(b) All data retrieved from the UN Comtrade Database at https://comtrade.un.org/data/.

**TABLE 2:** Import dependence of Middle Eastern countries from Russia and Ukraine (2016–2020) in key food categories.

Country^a, b^	Import dependence ratios from Ukraine (U) and Russia (R) total imports value in billion USD dollar (Tot) and percentage from U or R (%)											
	Cereals (Tot)	Cereals%		Milling industry (Tot)	Milling industry%		Oil seeds (Tot)	Oil seeds%		Fats and oil (Tot)	Fats and oil%	
		*R*	*U*		*R*	*U*		*R*	*U*		*R*	*U*
Algeria (16–17)	5.5	1.20%	2.70%	0.05	0.90%	0.02%	0.05	0.90%		1.6	23%	5%
Bahrain (16–19)	0.4	0.10%	0.20%	0.0081	0.01%	0.16%	0.05%	0.70%	0.20%	0.182	0.10%	1.10%
Egypt (16–20)	23	34%	24%	0.163	0.10%	6%	7.9	0.30%	12%	6.4	14%	4.40%
Iran (16—18)	10.6	7.30%	3.20%	0.02%	NA	6.50%	4.4	1%	3.70%	3.2	12%	9%
Israel (16–20)	4.4	8%	16%	0.4	2%	6%	1.9	0.10%	3.20%	0.9	4%	6%
Jordan (16–20)	3.9	14%	7.40%	0.1	0.30%	0.50%	0.6	0.30%	0.50%	0.89	2.20%	20%
Kuwait (16–20)	2.9	2.60%	1.70%	0.024	0.10%	0.40%	0.25	0.10%	0.40%	0.26	0%	1%
Lebanon (16–20)	1.6	22.60%	30.40%	0.17	0.09%	5.20%	0.485	0.02%	4.30%	0.7	16%	30%
Libya (16–18)	1.8	15%	35%	0.09	NA	4.30%	0.07	NA	4%	0.74	NA	1.40%
Mauritania (16–20)	0.9	14.50%	19.80%	0.01	NA	NA	0	NA	NA	0.06 (R); 0.16 (U)	0.01% ^8^	0.08% ^9^
Morocco (16–20)	8.7	5.50%	13.40%	0.02 (R); 0.08 (U)^10^	0.19%	2%	0.911	0.30%	1.50%	1.8	1.80%	1.40%
Oman (16–18)	1.3	14%	1.50%	0.0412	0.03%	6.50%	0.0213	0%	0.20%	0.2314	3.60%	6.80%
Qatar (16–20)	1.4	10.40%	7.80%	0.115	0.30%	3.10%	0.0716	0.04%	0.20%	0.5 (R);0.09 (U)^17^	0.07%	7.80%
Saudi Arabia (16–20)	17	7%	7%	0.23 (R);1.14 (U)^18^	0.09%	0.50%	0.93 (R); 4.2 (U)^19^	0.03%	0.06%	4.3	4.60%	5.10%
State of Palestine (16–20)	0.63	3.80%	0.60%	0.32	1.30%	33%	0.0921	0.13%	0.17%	0.25	2.70%	20.70%
Sudan (16–18)	2.422	76%	1.70%	0.423	7.30%	0.20%	0.07	NA	NA	0.7	23%	19%
Tunisia (16–19)	3.2	5.60%	40%	0.0224	0.04%	0.90%	0.925	0%	1.30%	0.9	15%	5%
Turkey (16–20)	12.5	51%	12%	0.64	11%	3%	10.3	9%	17%	7.3	30%	6.30%
United Arab Emirates (16–20)	6.1	10%	1.30%	0.6	0.17%	3.90%	4.95	0.02%	1.75%	2.8	0.50%	11%
Yemen (18–19)	1.5	20.60%	10.60%	0.18	NA	NA	0.02	NA	NA	0.08	NA	0.77%

(a) The years for calculated averages indicated after country name; e.g., 16–20 for years 2016–2020. If the years for the available data differ from the given years after the country name, this is indicated through number annotation as follows: ^1^ 2016 only; ^2^ 2017–2019; ^3^ 2016, 2017, 2019, and 2020; ^4^ 2020 only; ^5^ 2018–2020; ^6^ 2017 only; ^7^ 2018 only for Russia, and 2020 only for Ukraine; ^8^ 2018 only; ^9^ 2020 only; ^10^ 2019 only for Russia and 2016–2020 for Ukraine; ^11^ 2018–2020; ^12^ 2016 only; ^13^ 2016 only; ^14^ 2017 only; ^15^ 2016–2018; ^16^ 2016–2017; ^17^ 2020 only for Russia and 2016–2020 for Ukraine; ^18^ 2020 only for Russia and 2016–2020 for Ukraine; ^19^ 2020 only for Russia and 2016–2020 for Ukraine; ^20^ 2016–2019; ^21^ 2019–2020; ^22^ 2017–2018; ^23^ 2016–2017; ^24^ 2019 only; ^25^ 2017–2019.

(b) All data retrieved from the UN Comtrade Database at https://comtrade.un.org/data/.

## 4. Results

### 4.1. Assessing baseline vulnerability and food dependence

In order to determine the vulnerability of Middle Eastern countries to the current shock related to the Ukraine war, one needs to assess the relative importance of any potential disruptions in the components of the food value chain related to Ukraine and Russia. Firstly, the importance of food trade between Russia, Ukraine, and the Middle East can be shown using key categories of food commodities. Table 1 shows the biggest importers from Russia and Ukraine in the last 5 years (2016–2020), identifying some Middle Eastern countries as important trade partners of Russia and Ukraine. Egypt ranks highly as the biggest importer of cereals from both Ukraine and Russia, with a trade volume of more than 12 billion USD between 2016 and 2020. Turkey is also a key trade partner, particularly with regard to cereals, oil seeds, fats, and oil. Other Middle Eastern countries rank highly only in certain categories; notably, Iraq’s imports of fats and oil from Ukraine, or the UAE’s imports of milled products from Ukraine. In the category of cereals, ca. 30% of the trade value of Ukraine’s exports stems from eight Middle Eastern countries (Egypt, Turkey, Saudi Arabia, Tunisia, Israel, Libya, Iran, and Morocco). The top eight Middle Eastern countries for cereal exports from Russia (Egypt, Turkey, Iran, Saudi Arabia, Sudan, Yemen, Lebanon, and the UAE) account for ca. 40% of the total value of these exports between 2016 and 2020.

While the value of food exports from Russia and Ukraine to some countries might be relatively small, imports from Ukraine and Russia can still be high in terms of total imports. Table 2 shows the import dependence ratios of Middle Eastern countries from Russia and Ukraine using the value of these imports (data based on trade quantities largely not available). The data shows some dependence ratios of concern for several Middle Eastern countries, particularly in the categories of cereals and fats and oil. Note that data from some countries (Iraq, Djibouti, Syria, and Somalia) were not available, while some countries did not report consistent data for the period 2016–2020, or reported only the total values of all imports. Therefore, dependency ratios were calculated for the indicated years only, and a high dependence should only be assumed in the case of availability of data for several years and/or existence of high ratios across several indicators.

Tables 3, 4 seek to contextualize the dependence ratios by including a multi-dimensional food security factor to approximate the ability of a highly dependent Middle Eastern country to accommodate a supply interruption shock from Ukraine and/or Russia. This factor is represented by the Global Food Security Index (GFSI), which can indicate short- to mid-term food sector performance, and thus gives some information about the baseline vulnerability of a country. In determining this vulnerability to the Ukraine war shock, this paper combines the dependence ratios with the GFSI scores (Table 4). Here, countries with dependence ratios below 10% in all categories are not considered vulnerable: i.e., Bahrain and Kuwait. The Group 1 countries show some level of vulnerability that is not necessarily threatening due to a low level of dependence (10–20%) and/or quite high food sector performance (GSFI above 75%). This paper will focus on the case study analysis on Groups 2–3 of moderately to highly vulnerable countries, which indicate dependence ratios of above 20%, together with a poorly developed food sector (GFSI below 75%). In addition, Group 4 of special cases will be mentioned in the discussion of the results but not analyzed in detail.

**TABLE 3:** Ranking of Middle Eastern countries in the Global Food Security Index (GFSI) 2021.

Country ^a,^ ^b^	Overall GFSI score (and rank)	Score (and rank) in the subcategory “affordability”	Score (and rank) in the subcategory “availability”	Score (and rank) in the subcategory “quality and safety”	Score (and rank) in the subcategory “natural resources and resilience”
Algeria	63.9 (54)	77.9 (47)	58 (56)	62 (67)	50.7 (51)
Bahrain	68.5 (43)	79.2 (46)	67.5 (21)	79.9 (41)	39.1 (107)
Egypt	60.8 (62)	66.5 (68)	60.0 (49)	60.7 (71)	52.0 (44)
Israel	78 (12)	90.6 (7)	75.2 (6)	90.07 (10)	47.6 (60)
Jordan	64.6 (49)	80.4 (42)	55.2 (64)	63.5 (64)	54.2 (36)
Kuwait	72.2 (30)	80.1 (44)	72.3 (12)	86.4 (20)	43.0 (93)
Morocco	62.5 (57)	75.1 (52)	51.8 (74)	72.3 (50)	49 (57)
Oman	70.0 (40)	88.8 (18)	57.3 (59)	83.8 (28)	45.2 (76)
Qatar	73.6 (24)	83.8 (31)	74.4 (9)	83.5 (29)	43.4 (91)
Saudi Arabia	68.1 (44)	75.0 (53)	67.8 (20)	79.8 (42)	44.3 (84)
Sudan	37.1 (110)	31.8 (107)	31.6 (109)	52.4 (85)	41.4 (99)
Tunisia	62.7 (55)	74.4 (56)	54.0 (66)	72.1 (53)	47.6 (60)
Turkey	65.1 (48)	67.6 (67)	61.6 (42)	75.8 (47)	56.4 (27)
UAE	71.0 (35)	75.9 (50)	71.3 (14)	88.8 (16)	43.6 (88)
Yemen	35.7 (112)	39.3 (96)	27.6 (112)	37.4 (108)	42.1 (96)

(a) Data for the Global Food Security Index (GFSI) 2021 available at https://impact.economist.com/sustainability/project/food-security-index/.

(b) The scores for the all GFSI indicators are between 0 and 100, while the rank indicated in () is among 113 countries (Rank 1 being the best rank).

Ranks up to 0.01 billion were rounded up (i.e., 0.045 = 0.01).

**TABLE 4:** Categorization of Middle Eastern countries in terms of vulnerability to food supply shocks from Russia and Ukraine.

Groups	Criteria and countries	
Group 1: Countries with a low level of vulnerability	Criterion 1: Ratio of import dependence (on Russia and Ukraine) from 10 to 20% in any category (see Table 2): *Iran, Oman, Morocco, Qatar, Saudi Arabia, United Arab Emirates*	Criterion 2: Import dependence ratio above 20% in any category but with a high GFSI above 75/100: *Israel*
Group 2: Moderately vulnerable countries	Criterion 3: Import dependence ratio of more than 20% in any category but with a moderate GFSI from 50 to 75/100: *Egypt, Jordan, Tunisia, Algeria, Turkey*	
Group 3: Highly vulnerable countries	Criterion 4: Import dependence ratio in any category of more than 20% but GFSI below 50/100: *Yemen, Sudan*	Criterion 5: Import dependence ratio in any category more than 50% but not ranked in the GFSI: *Lebanon, Libya*
Group 4: Special or unclear cases	Criterion 6: Import dependence ratio in any category from 20 to 50% but not ranked in the GFSI: *Mauritania, Palestine.*	

### 4.2. Moderately vulnerable countries: Political-economic context and state-led responses

#### 4.2.1. “Manageable” pressures and balanced responses: Algeria, Jordan, and Turkey

The group of moderately vulnerable countries contains some Mediterranean countries with relative resilience and varying levels of dependence on imports from Ukraine and Russia. The level of dependence on Ukraine’s agricultural exports matters more since it has become a main war area. This can divide Group 2 countries into two subgroups, with Algeria, Jordan, and Turkey forming the first group with “manageable” pressures, since their dependence levels are not very high and rather skewed toward Russia rather than Ukraine. In this category, Algeria has a concerning (more than 20%) dependence on Russia only in the category of oils and fats. As a result of the Ukraine war, Algeria has suffered from double-digit inflation, particularly hitting food staples whose prices were liberalized in 2021 through the removal of food and energy subsidies (37). However, the inflation and the removal of the food subsidies have been common to other Middle Eastern countries. Recently, some food subsidies were replaced with social safety programs in Egypt, Mauritania, Algeria and Sudan, and these programs targeting the poor can mitigate some of the potential impacts of the Ukraine war (9). Besides, Algeria does not show dependence on Russia or Ukraine regarding grain, which is imported from France (38). Algeria, the third largest wheat importer in the world, has for a long time disallowed the import of Russian wheat (39). Furthermore, as a significant gas-exporting country, rising gas prices can help mitigate some of the food-related impacts in Algeria, or reduce its high dependence on oils and fats from Russia.

In Jordan, some limited level of dependence exists, mostly on Russia regarding cereals and oils, but it can be mitigated for this small-sized country (ca. 10 million inhabitants). Jordan has also mitigated past crises related to COVID-19 or the bans imposed by some countries on the exports of agri-food products. For example, in 2020, Romania banned wheat exports, thus triggering supply chain concerns across the Middle East (40). Although this ban only lasted for 6 days, it awakened some Middle Eastern countries such as Jordan, which imported one fifth of its cereals from Romania and needed to diversify its trade partners (41).

Similarly, in Turkey, dependence on cereals, the milling industry or fats and oil is strongly in favor of Russia. Particularly in cereals, Turkey–Russia trade flows are vitally important for Turkey, since Russia accounted for more 50% of the import value of cereals to Turkey in the last 5 years (Table 2). So far, Russia’s agricultural exports have not been directly targeted by sanctions, but some impacts in terms of rising prices are expected (42). At the same time, despite Russia accounting for a large amount of grains imported to Turkey, Turkey is largely self-sufficient in wheat and barley, while it exports processed wheat flour to other countries in the region, such as Iraq, Syria and Yemen. Turkey is the world’s largest wheat flour exporter (39). Turkish–Russian food relations remain important, and they have not suffered from the temporary bans on Russian grain exports, e.g., to ex-Soviet countries (43). As a result, Turkey is expected to mitigate the food crisis through a range of measures focusing on domestic markets; e.g., increasing domestic production, export bans, and aid to vulnerable groups, including the large population of Syrian migrants (44, 45). Besides, Turkey – with the United Nations – brokered the BSGI in mid-2022, and it has since then been one of the main destinations for Ukrainian shipments and grains (receiving more than 2 million tons out the 17.8 million tons of grains shipped from Ukraine after the BSGI as of the 18th of January 2023) (17).

#### 4.2.2. High exposure and long-term supply reorientation: Egypt and Tunisia

**FIGURE 1:**
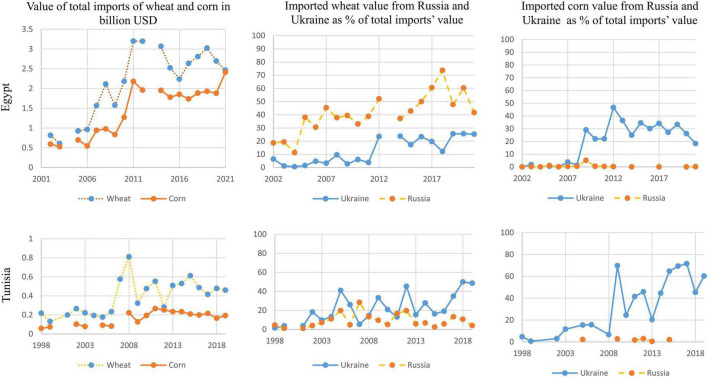
Imports to Tunisia and Egypt of wheat and corn from Russia and Ukraine [Data from UN Comtrade database for the commodity codes 1,001 “Wheat and meslin” and 1,005 “Maize” (corn)].

Both Egypt and Tunisia exhibit high dependence on Russia and Ukraine for the import of cereals, oil seeds (in the case of Egypt) and fats and oil (see Table 2). The high value of cereal imports from Ukraine is concerning, particularly in the case of Tunisia. However, Tunisia is a much smaller country (ca. 12 million in comparison to Egypt’s 102 million) with relatively stable food demands due to a smaller population growth rate; e.g., Tunisia’s population grew in the last 20 years (2001–2020) by 1% on average in comparison to 2% in Egypt for the same period (calculated from data.worldbank.org). This demographic difference is also shown in Figure 1 using the key grains of wheat and corn with data on the import values from the UN Comtrade. Quantity data (using kg of imports) are less available, but they differ only slightly with regard to the percentages of imports from Russia and Ukraine. For Tunisia, despite stable imports (particularly of corn), there is a high dependence on Ukraine for the import of wheat, and even higher for corn. However, these dependence rates have significantly fluctuated over the years. In contrast, Egypt, a country of markedly rising demands and imports over the last 20 years, relies more on Russia for wheat, but percentages of imports from Ukraine for both wheat and corn have been stable or decreasing in recent years.

There are important impacts and long-term implications for Tunisia and Egypt. For Tunisia, there are compounded impacts from COVID-19, recent political turmoils, and the Ukraine war. The rising costs of food imports, fuel, and fertilizers will weigh heavily on the Tunisian economy. Since food subsidies are substantial, the higher costs could add 1.5 billion USD to the subsidy bill (46). This comes after COVID-19 caused an economic decline in Tunisia, promoting the government to ramp up social transfers and support to businesses (47). Tunisia has been undergoing a political crisis after the dismissal of the government and the freezing of parliament in July 2021; parliament was dissolved by the President in March 2022. Meanwhile, it is struggling to curb food inflation, finance its increasing subsidy bill, and certify new food suppliers before its storage capacity runs out. Tunisia has developed some grain reserve capacities with the aim of having a national storage capacity of over 6 months for wheat (48). In early 2022, the government stated that the grain reserves will last until May 2022, but there were doubts about the impact of these reserves on food availability (49). Tunisia has been a recipient of some shipments from under the BSGI (50). However, food price hikes and fuel shortages continued during 2022, while the country political crisis has worsened by the end of the same year (51).

Egypt has been exploring long-term reorientation through new sources such as India (52). While India announced the halting of its wheat exports in May 2022, Egypt had already secured some shipments prior to this announcement. Similarly, to Tunisia, Egypt will see its subsidy bill increase significantly (53). While it has some grain reserves until the end of the year, China can also help Egypt, the world’s biggest wheat importer, by exporting wheat from the its huge grain stockpile (54). Egypt has also placed emphasis on support to local grain production. Table 5 compares wheat and corn production and import volumes in the Middle Eastern countries analyzed, indicating that both Egypt and Tunisia have strong domestic wheat and corn (in the case of Egypt) capacities. These domestic productions can help mitigate some long-term implications of the Ukraine war, although self-sufficiency seems difficult considering the consistently rising demands, especially in Egypt. Despite the local wheat markets suffering from the COVID-19 pandemic (55), Egypt has expanded its wheat production with the recently expanded Toskha project in the South Valley in Aswan expected to markedly increase wheat production. Egypt expects to get four millions of tons of wheat during its local harvest starting in April 2023 (56). As for the rising subsidy bill, Egypt has sought the help of the IMF to alleviate short-term funding pressures, while its GCC partners (Saudi Arabia, the UAE and Qatar) have allotted a total of 23 billion USD of investment in Egypt – some of which is going to Egypt’s central bank for assistance with food subsidies (57). Egypt’s has been one of the main recipient countries of the BSGI (receiving ca. 683.000 tons of grains until late January 2023) (36). However, the pressure on wheat prices did not ease due to fiscal difficulties (including a currency devaluation of nearly 50% since March 2021), and a shortages of foreign currency leaving hundreds of thousands of tons of wheat stuck at ports in late 2022 (56).

**TABLE 5:** Wheat and corn production and imports in selected Middle East countries.

Country	Wheat production (P)^a^ and imports (I)^b^ in million tons						Maize (Corn) production (P)^a^ and imports (I)^b^ in million tons					
	2000 P	2000 I	2010 P	2010 I	2020 P	2020 I	2000 P	2000 I	2010 P	2010 I	2020 P	2020 I
Algeria	0.76	5.37	2.61	5.23	3.11	NA	0	1.48	0	2.78	0	NA
Egypt	6.56	4.9	7.18	9.93	9.00*	9.58	6.47	4.96	7.04	5.2	7.50*	8.51
Iran (Islamic Republic of)	8.09	6.58	12.14	1.41	15.00*	0.00^(18)^	1.12	1.18	1.66	3.63	1.40*	8.98^(18)^
Jordan	0.03	0.58	0.02	0.49	0.02 ^Im^	0.76	0.02	0.41	0.03	0.51	0.02 ^Im^	0.74
Lebanon	0.11	0.41	0.08*	0.51	0.14*	0.63	0	0.29	0.01 ^Im^	0.35	0.00*	0.56
Libya	0.13*	NA	0.13 ^Im^	1.06	0.13*	1.44^(18)^	0.01	NA	0.00*	0.31	0.00 ^Im^	0.75
Mauritania	0.00 ^Im^	0.09	0	0.32	0.01 ^Im^	0.69	0.01	0	0.02	0	0.02*	0.01
Morocco	1.38	3.44	4.88	3.24	2.56	5.52	0.1	0.9	0.28	1.9	0.03	2.87
Oman	0	0.27	0	0.25	0	0.70^(18)^	0.02 ^Im^	0.04	0.01 ^Im^	0.09	0.03 ^Im^	0.21^(18)^
Palestine	0.05	NA	0.02	0.07	0.03 ^Im^	0.04	0	NA	0	1.92	0	0.03
Qatar	0	0.04	0	0.14	0	0.06	0	0.01	0	0.02	0	0.1
Saudi Arabia	1.79	0.02	1.35	1.62	0.55	0.77	0.04	1.26	0.08	1.92	0.06	3.07
Sudan	0.21	0.75^(01)^	0.4	1.35	0.75	5.01^(18)^	0.05	0.03^(01)^	0.04	0.04	0.01	0.00^(18)^
Tunisia	0.84	1.39	0.82	1.91	1.04	1.85^(19)^	0	0.68	0	0.89	0	1.03^(19)^
Turkey	21	0.96	19.67	2.55	20.5	10.00^(19)^	2.3	1.29	4.31	0.45	6.5	4.35^(19)^
United Arab Emirates	0	1.07	NA	0.85	NA	1.26^(19)^	0.00 ^Im^	0.05	0.01	0.24	0.02 ^Im^	0.56
Yemen	0.14	NA	0.27	2.65	0.10 ^Im^	2.00^(19)^	0.05	NA	0.09	0.46	0.04*	0.70^(19)^

(a) All data of production are retrieved from the FAO stat https://www.fao.org/faostat/en/. Data annotated from FAO as follows: * = unofficial figure; Im = FAO data based on imputation methodology. All other data are official figures.

(b) All data retrieved from the UN Comtrade Database at https://comtrade.un.org/data/. NA = data not available. The following annotation applies if the year of the retrieved data differs from the indicated column year [(18) means year 2018].

### 4.3. Highly vulnerable countries: conflict-related context and aid interventions

#### 4.3.1. Political fragility and food security: Lebanon, Libya, and Sudan

The three countries share a relatively high import dependence but also a fragile political-economic context. One can argue that if it were not for political instability and associated economic troubles, Lebanon, Libya and Sudan would have been much better positioned to deal with any repercussions of the Ukraine war. For example, Libya is a resource-rich (considerable oil reserves) and small-sized country, which has been suffering from the aftermath of the 2011 Arab Spring. Similar to the carbon economies of the GCC, Libya has an arid climate with no significant domestic production (see Table 5). It has relied on its ability to provide food through state-managed cereal imports, storage, and subsidies. After the Ukraine war, Libya witnessed food price hikes (58, 59). The subsidy system, through a Price Stability Fund (PSF) controlled by the government as the cereal buyer, is no longer in place since 2011, while private mills are not able to import wheat without compensation (60). There were also doubts about the government’s claims regarding reserves lasting for 1 year (ibid.). The outcome of this food crisis remains open, while the only option for Libya seems to be the utilization of its oil revenues for sourcing new suppliers of cereals and reinstating a subsidy system. So far, the BSGI has proved important for mitigating some of the impacts since it provided Libya with more than 400 thousand tons of grains from Ukraine as of late January 2023 (36).

Lebanon has for a long time been one of the countries with the highest GDP per capita levels among non-carbon exporting countries in the Middle East. In recent years, Lebanon has suffered from political conflicts leading to a serious economic crisis including high inflation rates and a strong devaluation of the national currency, thus causing food insecurity (61). In the wake of the COVID-19 crisis, food insecurity increased to affect an estimated 36–39% of the adult population in 2022 (compared to 27% before the pandemic) (62). Although Lebanon has an important domestic wheat production sector, the rising cost of fuel and fertilizers in response to the Ukraine war has meant price spikes, adding to the woes of farmers already suffering from climate change and prolonged dry spells (63). With significant price increases (e.g., 25% of bread and 83% of sunflower oil in March 2021), the situation for Lebanon was dire, while the government has sought fresh imports from India, the USA, and Kazakhstan (64). The Ukraine war has not only affected food access in Lebanon, but it might have also increased unhealthy dietary patterns (65). However, importing from distant regions or decreasing import dependence through strengthening the agricultural sector in Lebanon might not decrease food costs, especially considering the economic and currency situation in Lebanon (66). At the same time, some countries such as India has already temporarily halted some exports (e.g., for wheat), except for to certain countries (e.g., Yemen). For Lebanon, aid partners such as France and Saudi Arabia have proved crucial, as they have committed to food-related projects, including for the large and vulnerable community of Syrian refugees (67). In early 2023, the European Union (EU) announced a support program of 25 million euro to help Lebanon fight food insecurity through immediate assistance and support to local agriculture (68). Besides, the BSGI was instrumental for Lebanon in holding and alleviating the food crisis for now (69).

Similarly, to Lebanon, international aid is a prominent short-term strategy for Sudan, which faces a serious hunger crisis, as food prices has been rising since 2021 due to domestic inflation, the dismantling of all forms of wheat subsidies in early 2022, and the fallout from the Ukraine war (70). Sudan’s dire situation comes despite the country holding one of the biggest arable land potentials in Africa and some of the world’s largest and oldest irrigation schemes (71, 72). While Sudan has a large agricultural output of corn, sorghum and other crops, it has been heavily reliant on Russia for its wheat production (Tables 2, 5). The current crisis in Sudan is aggravated by the political turmoil following the 2019 revolution and the 2021 military coup. Since then, inflation has been very high, with bread prices increasing tenfold between October 2021 and March 2022 (73). The increase of the price of fertilizers (mainly used for wheat production in Sudan) has also decreased the domestic wheat production (74). The increased food cost comes at a time when East Africa is facing what could be the worst drought in decades (75). As of January 2023, Sudan benefited from some grain shipments under the BSGI (ca. 65 thousand tons) (36), with a promise of more to come under a food humanitarian program to countries in Africa and Asia (76).

#### 4.3.2. Protracted conflicts and aggravated food insecurity: The tragic case of Yemen

Yemen has been one of the most publicized cases of a food insecurity emergency getting worse as a result of the Ukraine war. This is due to the compounded impacts of the current civil war since late 2014, the COVID-19 crisis and the dependence on cereal imports provided through international aid (77). The civil war has pushed people into poverty and hunger, and together with climate change impacts and the COVID-19 pandemic, Yemen is entirely reliant on food imports, with more than seven million people by the end of 2022 in the categories of “catastrophe” or “emergency” levels of hunger (78). The Ukraine war also comes at a time when aid agencies are suffering from shortfalls in funding (79). With around 80% of the 30 million Yemeni population dependent on aid, the United Nations (UN) attempted in March 2022 to raise 4.3 billion USD in aid for Yemen, but only 1.3 billion USD were promised (80). In April 2022, the United Nations reiterated the need to ramp up aid for the group of war-torn and vulnerable countries including Afghanistan, Yemen, and Syria (81).

The aggravated food insecurity in Yemen as a result of the Ukraine war is difficult to resolve in the short or medium terms. Yemen has been suffering from political fragility and recurrent political conflicts even since its independence in the mid-20th century (82). Moreover, despite having agricultural potential, decades of water over-abstraction, mismanagement, and cultivation of cash crops (including the widely used simulant *qat*) have left Yemen’s agricultural sector quite weak (83). Domestic production has also been negatively affected by the destructive role of the formal private sector. This sector promotes imports in alliance with state elites, makes unsustainable demands on water supplies, and lacks the interest or the will to invest in agriculture (84). As a result of the Ukraine war, feasible short-term remedies include food aid delivered through international organizations, and/or from the reserves of neighboring GCC countries, some of which (Saudi Arabia and the UAE) are involved in the current war. While a temporary ceasefire was announced in early April 2022, some observers saw the situation deteriorating if no lasting peace materializes and donors as well as neighbors do not increase their aid (85). As of January 2023, Yemen received grain shipments under the BSGI in the amount of 150 thousand tons (36). Besides, international aid, the continuation of the ceasefire and better agricultural conditions meant an improvement of food security situation in late 2022, but high levels of food insecurity still persist for millions of Yemenis (86).

## 5. Discussion: Contextual determinants and action priorities

Studying food insecurities in the Middle East in the wake of the Ukraine war shows the complexity of the crisis, since it is accompanied by a set of internal and external aggravating factors. Unlike this recent crisis, previous food crises prior to the 2011 Arab Spring were related to poor harvests (e.g., in China), and thus spiking food prices; e.g., ca. 20% food inflation in Egypt in 2010–2011 (87). In the current crisis, both food supply disruptions and food price increases seem more significant, while they are accompanied by price increases in other vial commodities; e.g., fertilizers, fuel, and transport (88). Internally, this Ukraine-related crisis is hitting the Middle East very hard due to the relatively high dependence levels and the baseline political, economic, and environmental vulnerabilities. The BSGI and international aid have alleviated some of the impacts of the food security crisis in the region, but this crises is far from over as of early 2023. This paper has illustrated some of the specific contexts of the Middle East and the different levels of exposure to pressures associated with this crisis. Following from this, six contextual determinants of this exposure can be summarized:

(1) Existence of food subsidy or social security programs: Food subsidy systems or special programs providing food stamps for the most vulnerable have softened some of the impacts of the price hikes for vulnerable groups. However, in the Middle East, some countries (e.g., Algeria, Libya, Lebanon, and Sudan) have recently abandoned food subsidies, or have been unable to continue them due to political or economic difficulties. At the same time, the increased costs associated with these programs have caused fiscal difficulties, particularly in large countries such as Egypt.

(2) Cereal reserves and storage capacity: Although strategic grain reserves can be costly, some Middle Eastern countries have invested in such reserves in the aftermath of food crises in the last two decades (30, 31). Countries having significant storage capacity and available reserves were better able to avoid or delay shortfalls or price hikes despite high dependence (e.g., GCC countries and Egypt).

(3) Relative political-economic stability: Many of the most vulnerable countries in the current crisis are suffering from political conflicts and fragility (e.g., Libya, Lebanon, Sudan, and Yemen). Although this paper did not include Syria and Iraq in the analysis due to data availability, there are reports of similar food insecurities induced by the Ukraine war (23, 58). Political instability seems to be one of the factors influencing food insecurities in the aftermath of Ukraine war in other Middle Eastern countries not analyzed in this paper due to too specific a context; e.g., Palestine and Mauritania (89, 90).

(4) Baseline vulnerability to the COVID-19 pandemic: In some Middle Eastern countries such as Tunisia and Lebanon, the COVID-19 pandemic has greatly affected the states’ capacity to weather the current crisis, either due to reduced state revenues or aggravated food insecurities (47, 62). In other countries, evidence exists of indirect impacts through reduced yield (e.g., Egypt) (55), or the ramifications of regional political instability due to COVID-19; e.g., Sudan (27).

(5) Liquidity through additional revenues: In oil- and/or gas-exporting countries, the increase in carbon fuel prices after the Ukraine invasion meant additional revenues that can be used in the mitigation of the food crisis; e.g., in Algeria, Libya, or generally in the GCC countries. It is, however, not yet clear whether the additional revenues will be offset by the declining energy demand due to the global economic downturn.

(6) Existence of climatic aggravators: Recent dry spells, prolonged droughts and harvest failures can weaken the ability of local agriculture to provide food, as some narrative evidence from Sudan and Lebanon indicates (33, 63, 75).

With regard to responses in the Middle East, Table 6 summarizes the commonest interventions from this paper’s comparative analysis. These responses reveal how the Middle East is using indigenous solutions such as regional cooperation with the GCC countries positioned to play a central role in alleviating some of the short-term pressures in quite vulnerable countries, particularly Egypt, Yemen, and Lebanon. It will also not be surprising to see increased regional assistance to Tunisia and Sudan if the food crisis persists. At the same time, multilateral efforts such as the BSGI have eased some of the pressure from this crisis in some highly vulnerable countries. In the long run, the Ukraine war invokes some of the lessons from the COVID-19 crisis regarding the need for special aid programs, fewer trade restrictions, and more sustainable and resilient local agriculture (27, 28, 91). At the same time, with the Middle East unlikely and undesiring (due to impacts on water) to achieve self-sufficiency, it is important to invest in strategic storage and the strengthening of the supply chain through (regional) cooperation (e.g., on trade or aid) (29, 30). At the same, a stronger collaboration between the state and the private sector, including transnational food companies and domestic private importers, has become more important for the Middle East in order to secure food commodities from the global value chain (92, 93). Encouraging sustainable consumption in order to manage food waste–e.g., Dubai’s initiative for food loss reduction–is also a valuable and cost-effective food security strategy on the long-run (94).

**TABLE 6:** Initial response countries to food insecurity in selected Middle East countries.

Response category	Intervention types	Country examples
Trade control and diversification	Bans on cereals exports; certification of new suppliers; brokerage of deals for new shipments; buy-outs from foreign stockpiles	Algeria, Egypt Jordan, Libya, Tunisia, Turkey
Support to domestic markets	Stabilization of fertilizers costs; incentives for local farmers; increased monitoring and anti-profiteering controls	Egypt, Tunisia, Turkey, Lebanon
International cooperation and aid programs	Emergency food aid (mainly through WFP); refinancing instruments through IMF; ramping up of social programs; direct support to organizations delivering food aid	Egypt, Lebanon, Sudan, Yemen
Regional cooperation mechanisms (through GCC states)	Investments in state companies; central bank deposits; direct food-related aid	Egypt, Yemen, Lebanon

## 6. Conclusion

The Ukraine war has unleashed a complex global food crisis with supply interruptions and rising costs of key agricultural inputs such as fuel, transport and fertilizers. Together with the climate-related impacts and the baseline vulnerabilities related to COVID-19 and various conflicts, many countries in the global South are paying a high price for basic food commodities such as cereals and cooking oil. To better understand the reach of such compounded food crises, the Middle East serves as an illustrative case related to a high dependence on food imports from Russia and Ukraine and a very difficult political-economic context. Even prior to COVID-19 and the current Ukraine-related crisis, Middle Eastern countries have suffered from repeated food shocks that have caused or exacerbated political crises and state collapse, and many of these countries were hard-hit by the food-related spillovers of the COVID-19 crisis. However, despite the importance of food imports from Russia and Ukraine for the region, not all Middle Eastern countries will be highly exposed to the food crisis in the wake of the Ukraine war.

Moderately vulnerable countries such as Algeria, Jordan and Turkey face “manageable” pressures due to lower levels of dependence, availability of alternative production domestically, or well-functioning food sectors. They also demonstrate the importance of food diplomacy in these countries in order to maintain the flow of vital cereals, e.g., Algeria with France, Turkey with Russia, or Jordan with Romania. Within the same group of moderately vulnerable countries, Egypt and Tunisia stand out as facing more exposure due to high dependence rates. However, Egypt and Tunisia have ramped up their food storage capacities in the past years and invested in expanding wheat infrastructure. Tunisia has had rather stable but fluctuating imports from Ukraine. In both cases, the exposure to the Ukraine-related food crisis is complicated by growing populations (Egypt), COVID-19 turbulence, and an increase in internal political conflicts (Tunisia). Some immediate exit strategies relied on securing additional funds (e.g., from Gulf states) for satisfying the soaring costs of food subsidies while re-orienting the food import strategies toward new sources in Asia. Later, the release of some shipments from Ukraine helped lower the supply pressure although economic difficulties and high food prices persisted.

The case of the group of highly vulnerable countries (Lebanon, Libya, Sudan and Yemen) will prove quite concerning. While one would expect countries such as Libya to funnel some of their (increased) oil revenues toward mitigating the new crisis, Lebanon, Sudan and Yemen will be relying on international cooperation in the short and medium term, including shipments under the BSGI. These countries illustrate how political-economic instability is aggravating the food crisis in the Middle East in the wake of the Ukraine war. Political-economic stability is one of six vulnerability determinants identified by this paper, which can be examined in the future through case studies that are more detailed. However, state responses can still play an important role in deciding the outcomes of the current crisis. Alongside classic responses such as trade controls, supply diversification, public support, and aid, this paper has argued that the relatively comfortable position of Arab Gulf countries will play a crucial role in mitigating some of the impacts on the Middle East through regional cooperation and aid-related food security and fiscal stability. With regard to future food security strategies, the Ukraine war has highlighted the importance of both domestic, regional and international resilience and adaptation measures. Enhancing local capacities in the areas of storage or the procurement of food supplies (e.g., through stronger public-private collaborations) will be important for mitigating future shocks. As previous crises have also shown (e.g., COVID-19), local agriculture remains an important food security tool in some Middle Eastern countries, but it should be securitized using sustainability and efficiency criteria (particularly the issues of water availability and use efficiency). While special aid programs from the international community toward the most vulnerable and conflict-ridden communities in the Middle East is essential for overcoming the current food crises, strengthening regional frameworks for collaboration on food security has emerged as an interesting long-term pathway for the Middle East region.

## Data availability statement

Publicly available datasets were analyzed in this study. This data can be found here: UN Comtrade Database https://comtrade.un.org.

## Author contributions

The author confirms being the sole contributor of this work and has approved it for publication.
